# Book Review: The Psychology of Meaning in Life

**DOI:** 10.3389/fpsyg.2021.720828

**Published:** 2021-08-04

**Authors:** Yonghe Ti, Jiangfeng Yang

**Affiliations:** ^1^Institute of Education, Tsinghua University, Beijing, China; ^2^College of Education, Zhejiang University, Hangzhou, China

**Keywords:** meaning in life, crises of meaning, existential indifference, meaning in work, interventions

Several questions may emerge in people's minds sometime in their course of life: “What kind of life am I living?” “What do I struggle for?” “Why do they worth occupying my life?” When people encounter these questions, they might query the current condition indignantly, find themselves at a loss, or even choose to escape from reality. “Meaning becomes conscious and can be experienced but only in the form of its absence” (p. 35). This book, authored by Schnell (2009), investigates meaningfulness comprehensively and creates a chance for readers to introspect and explore their inner meaningfulness.

## Evaluation of the Book's Content

Since the author is a brilliant psychologist, the structure and arrangement of contents in this book seem to depend on the author's own experience and sensibility of research on meaning in life. The author avoids just listing the outcomes of a series of studies but composes the vivid instances and stories insightfully to assist the readers to explore the meaningfulness.

There are 14 chapters in this book. After attracting readers' attention by arousing the calling for meaning in modern society in Chapter 1, this book depicts the panorama centered on the concept of meaningfulness in people's lives in the following eight chapters. “Meaning in life is a multidimensional concept (p. 6).” Chapter 2 clarifies the definitions of three concepts: meaningfulness, crises of meaning, and sources of meaning, respectively. Sequentially, Chapter 3 describes the quantitative and qualitative measurement of meaning in life. It also emphasizes the usability of Sources of Meaning and Meaning in Life Questionnaire (SoMe; Schnell, 2009) and SoMe Card Method (La Cour and Schnell, 2020) for capturing one's states of meaning. Chapter 4 exemplifies the elements of the meaning pyramid for individuals, and Chapter 5 illustrates the varieties and dynamics of meaning in people's life course. To remind readers of reviewing their potential sources of meaning, Chapter 6 introduces the predictors of meaning and three critical characteristics of sources of meaning: breadth, balance, and depth. Then, the relationships between social inclusion and meaning are discussed in Chapter 7. For those who undergo low meaning, crises of meaning (Chapter 8) and existential indifference (Chapter 9) are shown and explained meticulously and discreetly.

The rest chapters of this book contain various topics related to meaningfulness in life practice. Does happiness mean meaningfulness? The author answers this question by distinguishing the concepts of eudaimonism from hedonism and accounts for the resonated story of Frankl to explicate that meaningfulness has a unique value for humans (Chapter 10). Because meaningfulness is conducive to mental and physical health (Chapter 11), this book also exemplifies existential psychological interventions for patients to promote their meaningfulness in Chapter 12. Faced with the fact that work is inseparable from modern life but usually makes people exhausted and depressed, Chapter 13 discusses the meaning in work. It reveals that the meaning of work is not equivalent to the sources of meaning for individuals. In the last chapter, the author ends up with a further brief outlook on meaning in life under modern society, which echoes this book's start.

## Discussion

Each chapter in this book tries to lead readers by questions, and these queries help disclose the mystery of meaningfulness step by step. The self-exploration part based on the previous content at the end of each chapter is ingenious and remarkable. Readers can grasp helpful tips or experience simple activities to look into their own life and promote their subjective well-being. These activities, such as filling the personalized model of meaning (p. 36) and reviewing life as a book exercise (p. 199), are also convenient for clinical staff to help people clarify their current condition and find their directions illuminated by their heart.

On the whole, this book transcends the simple synthesis of psychological foundations of meaning in life. As the book suggests, modern society offers people the freedom to capture their diverse meaning while social structure overwhelmingly isolates their underlying sources of meaning. Then it is easier for students and workers who are desperate for autonomy to complain about the aimlessness and meaninglessness of their daily lives. This book reveals the possible underlying causes of the emergence of “invisible youth” (p. 126) and “downshifting” (p. 226) from the angle of crises of meaning and existential indifference. It suggests that educators and clinical practitioners should tolerate the condition and make people aware that life deserves meaningfulness. Living a generative life, paying more attention to the common interest, and making the welfare more connected with others are the intrinsic thematic appealing of this book.

Although this book is valuable and helpful, there are also some demerits that potential readers should know. First, this book lacks the space to systematically indicate the complex process of searching for meaning, which is also an inevitable issue in the psychology of meaning in life (Steger et al., 2008a,b). Besides, the empirical evidence of this book is mainly based on the Western people. Nevertheless, cultural differences may cause a different understanding of meaning in life (Steger et al., 2008a). Last but not least, there are too many incomplete words that are probably resulted from print mistakes in this book, which bothers the fluent reading a lot. If the second edition would be considered, the author could improve this book from these aspects.

Overall, this book can provide students, psychological scholars, and professional clinical practitioners with the elementary psychological knowledge of meaningfulness and serve as a guide for those who are confused with or interested in their life meaning to self-reflect and self-educate.

## Author Contributions

YT wrote the manuscript. JY revised it. All authors contributed to the article and approved the submission for publication.

## Conflict of Interest

The authors declare that the research was conducted in the absence of any commercial or financial relationships that could be construed as a potential conflict of interest.

## Publisher's Note

All claims expressed in this article are solely those of the authors and do not necessarily represent those of their affiliated organizations, or those of the publisher, the editors and the reviewers. Any product that may be evaluated in this article, or claim that may be made by its manufacturer, is not guaranteed or endorsed by the publisher.
